# PINK1‐mediated mitophagy maintains pluripotency through optineurin

**DOI:** 10.1111/cpr.13034

**Published:** 2021-05-01

**Authors:** Chaoqun Wang, Kun Liu, Jiani Cao, Liang Wang, Qian Zhao, Zheng Li, Honghai Zhang, Quan Chen, Tongbiao Zhao

**Affiliations:** ^1^ State Key Laboratory of Stem Cell and Reproductive Biology Institute for Stem Cell and Regeneration Institute of Zoology Chinese Academy of Sciences Beijing China; ^2^ School of Life Sciences Qufu Normal University Qufu China; ^3^ Savaid Medical School University of Chinese Academy of Sciences Beijing China; ^4^ Department of Digestive System Beijing Tiantan Hospital Capital Medical University Beijing China; ^5^ College of Life Sciences Nankai University Tianjin China

**Keywords:** embryonic stem cells, mitochondria, mitophagy, OPTN, PINK1, reprogramming

## Abstract

**Objectives:**

Dysfunction of autophagy results in accumulation of depolarized mitochondria and breakdown of self‐renewal and pluripotency in ESCs. However, the regulators that control how mitochondria are degraded by autophagy for pluripotency regulation remains largely unknown. This study aims to dissect the molecular mechanisms that regulate mitochondrial homeostasis for pluripotency regulation in mouse ESCs.

**Materials and methods:**

*Parkin^+/+^* and *parkin* ^−/−^ ESCs were established from E3.5 blastocysts of *parkin^+/−^* x *parkin^+/−^* mating mice. The *pink1* ^−/−^, *optn* ^−/−^ and *ndp52* ^−/−^ ESCs were generated by CRISPR*‐Cas9*. shRNAs were used for function loss assay of target genes. Mito‐Keima, ROS and ATP detection were used to investigate the mitophagy and mitochondrial function. Western blot, Q‐PCR, AP staining and teratoma formation assay were performed to evaluate the PSC stemness.

**Results:**

PINK1 or OPTN depletion impairs the degradation of dysfunctional mitochondria during reprogramming, and reduces the reprogramming efficiency and quality. In ESCs, PINK1 or OPTN deficiency leads to accumulation of dysfunctional mitochondria and compromised pluripotency. The defective mitochondrial homeostasis and pluripotency in *pink1* ^−/−^ ESCs can be compensated by gain expression of phosphomimetic Ubiquitin (Ub‐S65D) together with WT or a constitutively active phosphomimetic OPTN mutant (S187D, S476D, S517D), rather than constitutively inactive OPTN (S187A, S476A, S517A) or a Ub‐binding dead OPTN mutant (D477N).

**Conclusions:**

The mitophagy receptor OPTN guards ESC mitochondrial homeostasis and pluripotency by scavenging damaged mitochondria through TBK1‐activated OPTN binding of PINK1‐phosphorylated Ubiquitin.

## Graphical abstract

Increasing reports show autophagy‐mediated mitochondrial homeostasis is essential for pluripotency regulation, but the receptors responsible for mitochondrial degradation in ESCs are unclear. Here, Wang et al identify OPTN is the mitochondrial autophagy receptor downstream of PINK1 that is responsible for regulation of mitochondrial homeostasis during both the acquisition and maintenance of pluripotency in a PARKIN‐independent manner.

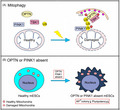

## 1 INTRODUCTION

Autophagy is a cellular degradation process that sequesters portions of cytoplasm into autophagosomes for degradation and recycling.^1^, ^2^, ^3^ In contrast to proteasome‐mediated degradation of small and short‐lived proteins, large protein aggregates and damaged organelles are degraded by autophagy.^4^ At first, autophagy was proposed to carry out bulk degradation of protein aggregates and organelles under stress conditions; however, more and more studies have provided evidence supporting the idea that autophagy is a selective process.^5^ Selective removal of mitochondria by autophagy, named mitophagy, has been extensively demonstrated. Mitophagy receptors bridge the damaged mitochondria with MAP1LC3B (LC3‐II) on autophagic membranes, leading to the cargo engulfment.^6^ Currently, two types of mitophagy receptors have been identified. One type, like p62/SQSTM1 and optineurin (OPTN), contain an ubiquitin‐binding domain that localizes them to ubiquitin‐tagged mitochondria. The other type, like BNIP3 and NIX, harbor a LC3‐II‐interacting region (LIR), and are located on mitochondria where they can be directly recognized by isolation membranes, which are precursors of autophagosomes.^4^

Pluripotent stem cells (PSCs), including embryonic stem cells (ESCs) and induced pluripotent stem cells (iPSCs), can undergo unlimited self‐renewal and give rise to any cells of the three germ layers; thus, they hold great promise for regenerative medicine.^7^, ^8^, ^9^, ^10^, ^11^, ^12^, ^13^ PSCs have distinct cellular components and organelles compared with somatic cells. Recent studies have demonstrated that mitochondria are remodeled by autophagy during reprogramming of somatic cells.^14^, ^15^ In ESCs, dysfunction of autophagy leads to accumulation of damaged mitochondria which thereafter inhibits self‐renewal and pluripotency.^1^, ^14^, ^16^, ^17^, ^18^ These studies indicate that mitochondria are strictly regulated by autophagy to maintain their homeostasis in PSCs. However, the molecules which are directly responsible for recognition of mitochondria by autophagy in ESCs have not been identified.

In this study, we showed that OPTN is a mitophagy receptor that regulates mitochondrial homeostasis in ESCs. OPTN deficiency, like PINK1 deficiency, leads to abnormal self‐renewal and reduced pluripotency of ESCs. PINK1 and OPTN are linked via PINK1‐mediated phosphorylation of Ub and TBK1‐mediated activation of the Ub‐binding function of OPTN in a pathway that is independent of the E3 ligase PARKIN.

## 2 MATERIALS AND METHODS

### 2.1 Reagents, antibodies, and plasmids

TMRE (AAT Bioquest, 22 220), MitoTracker Green (Yeasen, 40742ES50), and DCFH‐DA (Sigma Aldrich, D6883) were used to examine mitochondrial function. The following antibodies were used: anti‐PINK1 polyclonal antibody (NOVUS, BC100‐494), anti‐OPTN polyclonal antibody (Proteintech, 10837‐1‐AP), anti‐NDP52 polyclonal antibody (Proteintech, 12229‐1‐AP), anti‐UB monoclonal antibody (Santa Cruz Biotechnology, sc‐8017), anti‐ACTIN monoclonal antibody (Sigma Aldrich, A5441) SSEA‐1 (Santa Cruz Biotechnology, SC‐21702AF647). Reprogramming factors pMXs‐*Pou5f1* (Addgene, 13 366), pMXs‐*Sox2* (Addgene, 13 367), pMXs‐*Klf4* (Addgene, 13 370), and pMXs‐*cMyc* (Addgene, 13 375) were deposited by Shiya Yamanaka's lab. *Pink1*, *Pink1* mutants,^19^, ^20^ *Parkin*, *Optn*, and *Optn* mutants were cloned into pMXs, pCDH‐CAG‐PURO and pCDH‐CAG‐RFP lenti‐vectors as described previously. ShRNAs were designed and cloned into pSicor‐GFP and pSicor‐Vector.

### 2.2 ESC isolation and knockout ESC generation

*Parkin^+/+^* and *parkin* ^−/−^ ESCs were isolated at day E3.5. The inner cell mass was picked and cultured in 2i medium. The early‐passage ESCs were cultured in 2i medium, and the later passages were maintained in ESC medium. Medium was used as described previously.^14^

*Pink1*, *Optn* and *Ndp52* knockout ESCs were generated by CRISPR‐Cas9. We designed the gRNAs to target *Pink1*, *Optn* and *Ndp52*, and transfected the gRNA vector into ESCs using a Gene Pulser Xcell II (Bio‐Rad) according to the manufacturer's protocols. Knockout ESCs were identified by sequencing and western blotting.

### 2.3 Somatic cell reprogramming

Around 50 000 MEFs per well were seeded into 6‐well plate and infected with reprogramming vector cocktails (Pou5f1, Sox2, Klf4 and c‐Myc) as previously described.^9^ 24 hours after infection, the medium were changed with fresh ESC medium and replaced every day. Colonies occurred approximately 12 days after reprogramming.

### 2.4 Western blot analysis

Cell samples were lysed in RIPA buffer (50 mM Tris‐HCl, pH 7.4, 150 mM NaCl, 0.5% sodium deoxycholate, 1% Nonidet P‐40, 5 mM EGTA, 2 mM EDTA, 10 mM NaF) with protease inhibitor cocktail (Roche, 04693116001). Equal quantities of protein were loaded onto gels for SDS‐PAGE, and transferred to nitrocellulose membranes (Millipore). The first antibody was used at the indicated concentration to incubate the membrane. Then the membrane was incubated with an appropriate HRP‐conjugated secondary antibody (Santa Cruz). A Luminata Forte Western HRP Substrate Kit (Millipore, WBLUF0100) was used to detect the immunoreactive bands.

### 2.5 Mito‐Keima assay

The Mito‐Keima constructs were employed to monitor mitophagosome formation as previously described.^21^ The cells under normal conditions or treated with FCCP were detected by a confocal laser scanning microscopy for Mito‐Keima imaging.

### 2.6 AP staining, clone formation assay, and measurements of mitochondrial mass, ROS and ATP

A BCIP/NBT Alkaline Phosphatase Colour Development Kit (Beyotime, C3206) was used for AP staining according to the manufacturer's instructions. For the clone formation assay, 1000 ESCs were seeded onto feeder cells and cultured for a week. The number of AP‐positive colonies was counted after AP staining. To measure the mitochondrial mass, cells were incubated in medium with 100 nM MitoTracker Green at 37°C for 30 min, then the mitochondrial mass was determined using a FACSCalibur flow cytometer. HDCF‐DA was used for measuring ROS by flow cytometry. Cellular ATP content was detected using a Cell Titer‐Glo Luminescent Cell Viability Assay kit (Promega Corporation, 0000092970). All the details are as described previously.^14^

### 2.7 Quantitative real‐time PCR

Total RNA was extracted using an RNeasy Total RNA Isolation Kit (Qiagen, 74 104), then reverse transcribed into cDNA using a SuperScript™ III First‐Strand Synthesis System (Invitrogen Thermo Fisher Scientific, 18 080 051). Quantitative real‐time PCR was performed with GoTaq® qPCR Master Mix (Promega, A6002) and a LightCycler 480 Real‐Time PCR System (Roche). The following primers were used: *Pou5f1*: 5′‐AGAGGATCACCTTGGGGTACA‐3′ (forward), 5′‐CGAAGCGACAGATGGTGGTC‐3′ (reverse); *Nanog*: 5′‐TCTTCCTGGTCCCCACAGTTT‐3′ (forward), 5′‐GCAAGAATAGTTCTCGGGATGAA‐3′ (reverse); *Sox2*: 5′‐GCGGAGTGGAAA CTTTTGTCC‐3′ (forward), 5′‐CGGGAAGCGTGTACTTATCCTT‐3′ (reverse); *Esrrb*: 5′‐CAG GCAAGGATGACAGACG‐3′ (forward), 5′‐GAGACAGCACGAAGGACTGC‐3′ (reverse); *Rexo1*: 5′‐CCCTCGACAGACTGACCCTAA‐3′ (forward), 5′‐TCGGG GCTAATCTCACTTTCAT‐3′ (reverse); *Cdh1*: 5′‐CAGGTCTCCTCATGGCTTTGC‐3′ (forward), 5′‐CTTCCGAAAAGAAGGCTGTCC‐3′ (reverse); *Klf4*: 5′‐GTGCCCC GACTAACCGTTG‐3′ (forward), 5′‐GTCGTTGAACTCCTCGGTCT‐3′ (reverse); *Tcl1*: 5′‐AAATTCCAGGTGATCTTGCG‐3′ (forward), 5′‐TGTCCTTGGGGTACAGTTGC‐3′ (reverse); *Fbxo15*: 5′‐TCGTGGGACTGAGCACAACTA‐3′ (forward), 5′‐TGACAGATGAGCCT CTAACAAAC‐3′ (reverse); *Fgf5*: 5′‐CTGTATGGACCCAC AGGGAGTAAC‐3′ (forward), 5′‐ATTAAGCTCCTGGGTCGCAAG‐3′ (reverse); *Otx2*: 5′‐TATC TAAAGCAACCGCCTTACG‐3′ (forward), 5′‐ AAGTCCATACCCGAAGTGGTC‐3′ (reverse); *Scl*: 5′‐ CTGGCCTCCAGCTACATTTCT‐3′ (forward), 5′‐ GTCACGGTCTTTGCTCAACTT‐3′ (reverse); *Bra*: 5′‐GCTTCAAGGAGCTAACTAACGAG‐3′ (forward), 5′‐CCAGCAAGAAAGAG TACATGGC‐3′ (reverse); *Afp*: 5′‐CTTCCCTCATCCTCCTGCTAC‐3′ (forward), 5′‐ACAAA CTGGGTAAAGGTGATGG‐3′ (reverse); *Gata4*: 5′‐CCCTACCCAGCCTACATGG‐3′ (forward), 5′‐ACATATCGAGATTGGGGTGTCT‐3′ (reverse); *Actin*: 5′‐GGCTGTATTCCCCTC CATCG‐3′ (forward), 5′‐CCAGTTGGTAACAATGCCATGT‐3′ (reverse); *Nbr1*: 5′‐ GGAAATCAGCTACAGATGCAAGT‐3′ (forward), 5′‐ ATCCCAAGACTCTCACCAGTG‐3′ (reverse); *Ndp52*: 5′‐ GCCCCATACCTACCTTGCTG‐3′ (forward), 5′‐ TCGAGGGATGAACTTTTCAGTG‐3′ (reverse); *Optn*: 5′‐ ATGTCCCATCAACCTCTGAGC‐3′ (forward), 5′‐ TCAAATCGCCCTTTCATAGCTTG‐3′ (reverse); *Taxbp1*: 5′‐ TGCACACTTGGAGTGCCATTA‐3′ (forward), 5′‐ TGTTCAGGCATAGGAGACCATAA‐3′ (reverse); *P62*: 5′‐ AGGATGGGGACTTGGTTGC‐3′ (forward), 5′‐ TCACAGATCACATTGGGGTGC‐3′ (reverse); *Pink1*: 5′‐ CACACTGTTCCTCGTTATGAAGA‐3′ (forward), 5′‐ CTTGAGATCCCGATGGGCAAT‐3′ (reverse).

## 3 RESULTS

### 3.1 PINK1 regulates somatic cell reprogramming independently of PARKIN

**FIGURE 1:**
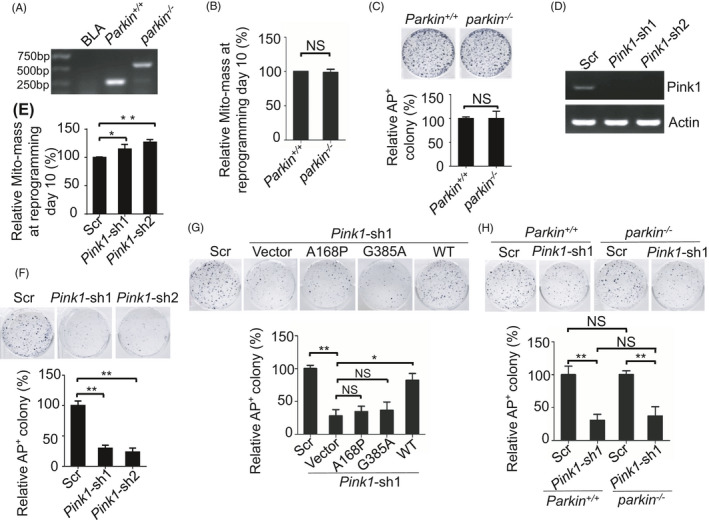
PINK1 but not PARKIN is essential for somatic cell reprogramming. A, Total genomic DNA extracted from *Parkin^+/+^* and *parkin* ^−/−^ MEF cells was amplified by PCR to confirm the knockout mutation in *Parkin*. BLA, H_2_O. B, PARKIN is dispensable for mitochondrial removal during reprogramming of somatic cells (MEFs). Cells stained with Mito‐tracker and anti‐SSEA‐1 antibody were detected by a FACS at reprogramming day 10. SSEA‐1‐positive cells were used to measure the Mito‐mass. Data are shown as mean ± SD, n = 3; NS, not significant; Student's *t* test. C, PARKIN is not required for somatic cell reprogramming. Colonies were stained with alkaline phosphatase (AP) on day 12 of reprogramming. Data are shown as mean ± SD, n = 3; NS, not significant; Student's *t* test. D, Cells were transfected with scramble short RNA or small interfering RNA targeting *Pink1*, then harvested and subjected to RT‐PCR analysis to detect *Pink1* mRNA expression. E, Inhibition of *Pink1* expression leads to mitochondrial accumulation during reprogramming. The Mito‐mass was determined in SSEA‐1‐positive cells using Mito‐RFP (which labels mitochondria with RFP) at reprogramming day 10. Data are shown as mean ± SD, n = 3; *P < .05; **P < .01; Student's *t* test. F, Knockdown of *Pink1* decreases the reprogramming efficiency. iPSC colonies were stained by AP at reprogramming day 12. Data are shown as mean ± SD, n = 3; **P < .01; Student's *t* test. G, The decreased reprogramming efficiency by *Pink1* knockdown was rescued by gain of expression of wild‐type but not kinase dead mutant (A168P or G385A) *Pink1*. Colonies were stained with alkaline phosphatase (AP) on day 12 of reprogramming. Data are shown as mean ± SD, n = 3; *P < .05; **P < .01; NS, not significant; Student's *t* test. H, While knockout of *Parkin* did not affect reprogramming efficiency, knockdown *Pink1* in both *Parkin^+/+^* and *parkin* ^−/−^ MEF cells significantly reduced reprogramming efficiency. Colonies were stained with alkaline phosphatase (AP) on day 12 of reprogramming. Data are shown as mean ± SD, n = 3; **P < .01; NS, not significant; Student's *t* test

PINK1/PARKIN‐mediated mitophagy is currently the best defined pathway that regulates mitochondrial homeostasis in somatic cells.^5^, ^22^, ^23^, ^24^ To test whether this pathway is involved in somatic cell reprogramming, we first investigated whether PARKIN regulates mitochondrial remodeling during reprogramming of mouse embryonic fibroblasts (MEFs). Knockout of *Parkin* did not affect mitochondrial clearance and reprogramming efficiency, which indicates that PARKIN‐mediated selective mitochondrial autophagy is not involved in mitochondrial remodeling during somatic cell reprogramming (Figure 1A‐C). We then investigated whether PINK1 is involved in somatic cell reprogramming. Knockdown of *Pink1* expression during reprogramming significantly inhibited mitochondrial clearance and colony formation, which indicates that PINK1 is essential for mitochondrial remodeling and acquisition of pluripotency for somatic cell reprogramming (Figure 1D‐F). Interestingly, the defective somatic cell reprogramming in *pink1* knockdown MEFs were rescued by gain expression of WT‐ but not kinase activity dead (A168P or G385A) mutant‐ PINK1, indicating the kinase activity of PINK1 is required for pluripotency acquisition (Figure 1G; Figure S1A). In addition, while knockout of *Parkin* did not change reprogramming efficiency, knockdown of *Pink1* expression in *parkin* ^−/−^ MEFs during reprogramming inhibited mitophagosome formation and significantly decreased reprogramming efficiency (Figure 1H; Figure S1B). Together, these data suggest that PINK1‐mediated mitophagy regulates mitochondrial remodeling and somatic cell reprogramming independently of PARKIN.

Furthermore, we established iPSCs with decreased *Pink1* expression (Figure S2A). We found inhibition expression of *Pink1* results in increased Mito‐mass, decreased mitochondrial membrane potential, elevated intracellular reactive oxygen species (ROS) and deteriorated ATP generation in established iPSCs (Figure S2B‐E). Correspondingly, the expression of pluripotency marker genes in *Pink1* knockdown iPSCs was significantly decreased compared to wild type (WT) iPSCs (Figure S6F). These data indicate inhibition of *Pink1* not only compromises reprogramming efficiency but also deteriorates iPSC quality.

### 3.2 PINK1 guards ESC identity

We next asked whether the PINK1/PARKIN pathway contributes to pluripotency regulation in ESCs. *Parkin^+/+^* and *parkin* ^−/−^ ESCs were isolated from mouse blastocysts at embryonic d 3.5 and were shown to have a normal karyotype (Figure S3A). Colony formation assays showed that the absence of *Parkin* did not affect ESC self‐renewal ability (Figure S3B). Furthermore, lack of *Parkin* did not change the expression of pluripotency genes, which indicates that PARKIN is dispensable for pluripotency maintenance of ESCs (Figure S3C).

**FIGURE 2:**
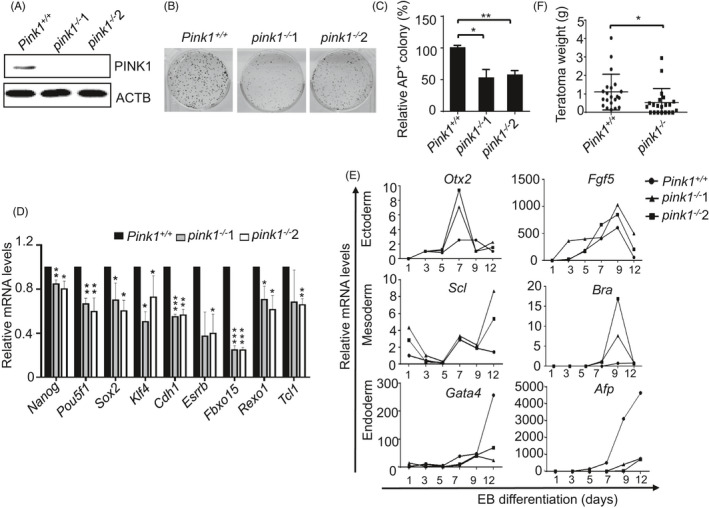
PINK1 maintains ESC identity. A, Western blot detection of PINK1 expression. Proteins extracted from *Pink1^+/+^* and *pink1* ^−/−^ ESCs were blotted and probed using anti‐PINK1 and anti‐ACTIN (ACTB) antibodies. ACTB serves as a loading control. B, Images of AP‐stained colonies formed by *Pink1^+/+^* and *pink1* ^−/−^ ESCs. *Pink1^+/+^* and *pink1* ^−/−^ ESCs were cultured on feeder layers for a week, and then stained with an AP staining kit. C, Statistical analysis of the colony numbers in B. Data are shown as mean ± SD, n = 3; *P < .05; **P < .01; Student's *t* test. D, Pluripotency gene expression in *Pink1^+/+^* and *pink1* ^−/−^ ESCs. Data are shown as mean ± SD, n = 3; *P < .05; **P < .01; ***P < .001; Student's *t* test. E, *Pink1* knockout results in delayed expression of endoderm marker genes. Total RNA was harvested from embryonic bodies (EB) at day 1, 3, 5, 7, 9 and 12 and the mRNA expression of the indicated genes was determined by RT‐PCR analysis. Data shown are representative of 3 independent experiments. F, *Pink1* deficiency leads to compromised teratoma formation. Data are shown as representative of 3 independent experiments

We then knocked out *Pink1* in ESCs by CRISPR/Cas9 (Figure 2A; Figure S4A‐C). We found that knockout of *Pink1* significantly inhibited the self‐renewal and pluripotency of ESCs (Figure 2B, C). Reduced colony formation of *pink1* ^−/−^ ESCs was not caused by abnormal cellular proliferation or apoptosis since *Pink1* knockout did not affect proliferation and viability (Figure S4D, E). Furthermore, *pink1* ^−/−^ ESCs showed significantly decreased expression of pluripotency genes, which suggests that knockout of *Pink1* leads to the compromised pluripotency in ESCs (Figure 2D). In support of this idea, *pink1* ^−/−^ ESCs showed abnormal embryonic body (EB) differentiation, characterized by delayed expression of certain endoderm marker genes and advanced expression of certain ectoderm genes (Figure 2E). In addition, the teratoma formation assay was employed to test the contribution of *Pink1* to ESC differentiation. While both *Pink1^+/+^* and *pink1* ^−/−^ ESCs formed teratomas, the average weight of teratomas formed by *Pink1^+/+^* ESCs is significantly higher than those formed by *pink1* ^−/−^ ESCs, which supports the notion that PINK1 is pivotal for differentiation of ESCs (Figure 2F). Taken together, these data indicate that PINK1 is essential for ESC self‐renewal, pluripotency and differentiation.

### 3.3 PINK1 is essential for maintaining mitochondrial homeostasis in ESCs

**FIGURE 3:**
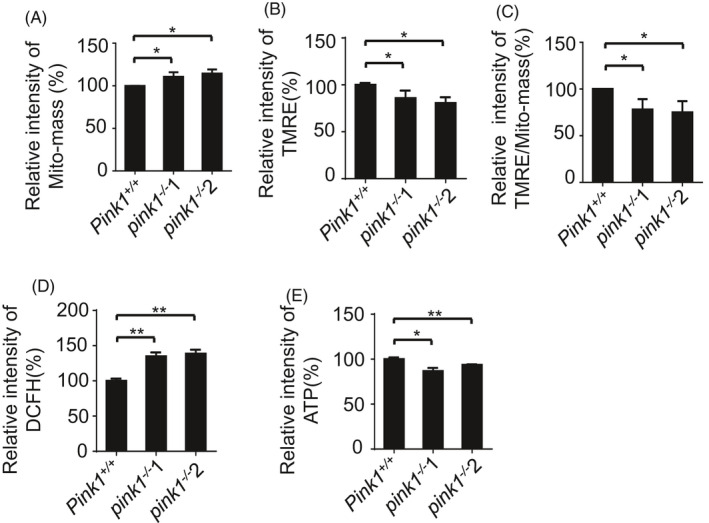
PINK1 regulates mitochondrial homeostasis in ESCs. A, Mitochondria accumulate in *pink1* ^−/−^ ESCs. The Mito‐mass was determined by a FACS using Mito‐tracker staining. Data are shown as mean ± SD, n = 3; *P < .05; Student's *t* test. B, Mitochondrial membrane potential decreases in *pink1* ^−/−^ ESCs. The mitochondrial membrane potential was detected in TMRE‐stained cells by a FACS. Data are shown as mean ± SD, n = 3; *P < .05; Student's *t* test. C, Relative mitochondrial membrane potential to Mito‐mass. Data are shown as mean ± SD, n = 3; *P < .05; Student's *t* test. D, Increased ROS generation in *pink1* ^−/−^ ESCs. The ROS level was detected in DCFH‐DA‐stained cells using a FACS. Data are shown as mean ± SD, n = 3; **P < .01; Student's *t* test. E, *pink1* ^−/−^ ESCs have a decreased ATP level. Data are shown as mean ± SD, n = 3; *P < .05; **P < .01; Student's *t* test

Since PINK1 is a critical regulator of mitochondrial autophagy in somatic cells, we next asked whether lack of PINK1 affects the mitochondrial homeostasis in ESCs. The mitochondrial mass was determined by a FACS using Mito‐tracker staining. We found that mitochondrial accumulation was higher in *pink1* ^−/−^ ESCs than in *Pink1^+/+^* ESCs (Figure 3A). Further investigation identified that depletion of *Pink1* significantly decreased the mitochondrial membrane potential and elevated the level of intracellular reactive oxygen species (ROS) (Figure 3B‐D). These data indicate that *Pink1* knockout leads to accumulation of damaged mitochondria in ESCs. As a result, *pink1* ^−/−^ ESCs generate less ATP than *Pink1^+/+^* ESCs (Figure 3E). Taken together, these data provide evidences that PINK1 regulates mitochondrial homeostasis to maintain normal physiological function in ESCs.

### 3.4 The autophagy receptor OPTN regulates mitochondrial homeostasis and pluripotency

Mitophagy receptors with ubiquitin‐binding activity, like OPTN, NBR1, NDP52, TAX1BP1 and SQSTM1, are targeted to depolarized mitochondria through the PINK1/PARKIN mitochondrial ubiquitylation pathway.^5^ Phosphorylation of the E3 ligase PARKIN by PINK1 activates PARKIN to assemble poly‐Ub chains on proteins located in the outer mitochondrial membrane (OMM) of damaged mitochondria.^23^ Furthermore, PINK1 becomes stabilized on damaged mitochondria, and can recruit OPTN to activate mitophagy independently of PARKIN.^22^ Phosphorylation of OPTN on Serine 473 (S473) by Tank‐Binding Kinase 1 (TBK1) enables binding of OPTN to Serine 65 (S65)‐phosphorylated ubiquitin in PINK1‐driven and PARKIN‐independent mitophagy.^25^ To determine the regulators in this pathway that are responsible for pluripotency regulation, we screened the effects of knocking down OPTN, NBR1, NDP52, TAX1BP1 and SQSTM1 (p62) on somatic cell reprogramming. Interestingly, among these receptors, knockdown of *Optn* expression had the most significant inhibitory effects on reprograming efficiency (Figure S5A‐B). Correspondingly, inhibition of *Optn* expression leads to mitochondrial accumulation at reprograming day 10 (Figure S5C). In addition, although inhibition of *Ndp52* expression decreased reprogramming efficiency, knockdown of *Ndp52* in *Optn* knockdown MEFs during reprogramming did not enhance inhibitive effects of *Optn* knockdown (Figure S5D). These data indicate that OPTN contributes to mitochondrial remodeling and acquisition of pluripotency in somatic cell reprogramming.

**FIGURE 4:**
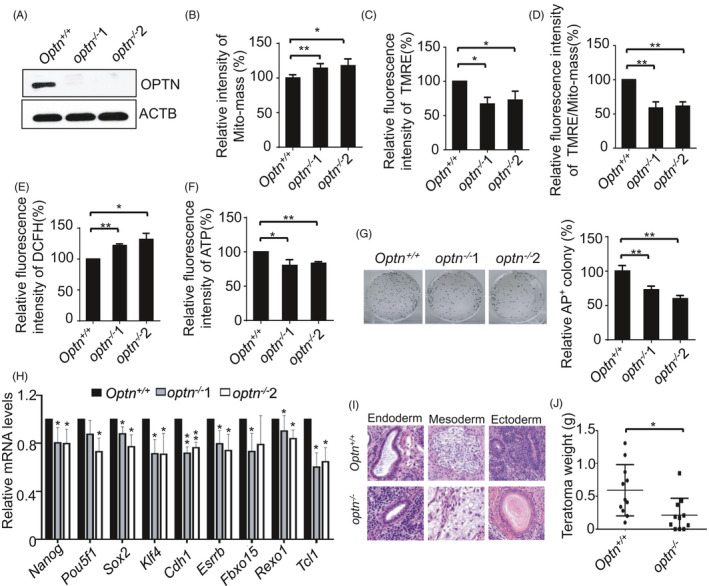
The autophagy receptor OPTN maintains mitochondrial homeostasis and pluripotency. A, Western blot detection of OPTN expression using anti‐OPTN and anti‐ACTB antibodies. ACTB serves as a loading control. B, Mitochondria accumulate in *optn* ^−/−^ ESCs. Data are shown as mean ± SD, n = 3; *P < .05; **P < .01; Student's *t* test. C, Mitochondrial membrane potential decreases in *optn* ^−/−^ ESCs. Data are shown as mean ± SD, n = 3; *P < .05; Student's *t* test. D, Relative mitochondrial membrane potential to Mito‐mass in both *optn^+/+^* and *optn* ^−/−^ ESCs. Data are shown as mean ± SD, n = 3; **P < .01; Student's *t* test. E, Enhanced ROS production in *optn* ^−/−^ ESCs. Data are shown as mean ± SD, n = 3; *P < .05; **P < .01; Student's *t* test. F, *optn* ^−/−^ ESCs generate less ATP than *optn^+/+^* ESCs. Data are shown as mean ± SD, n = 3; *P < .05; **P < .01; Student's *t* test. G, Knockout of *Optn* impairs self‐renewal of ESCs. Data are shown as mean ± SD, n = 3; **P < .01; Student's *t* test. H, Pluripotency gene expression in *optn^+/+^* and *optn* ^−/−^ ESCs. Data are shown as mean ± SD, n = 3; *P < .05; **P < .01; Student's *t* test. I, HE staining of teratomas formed by *Optn^+/+^* and *optn* ^−/−^ ESCs. J, Weight of teratomas formed by *Optn^+/+^* and *optn* ^−/−^ ESCs. Data are shown as mean ± SD, n = 3; *P < .05; Student's *t* test

We next investigated whether the autophagy receptor OPTN regulates mitochondrial homeostasis in ESCs and thereby affects ESC identity. *optn* ^−/−^ ESCs were generated by CRISPR/Cas9 (Figure 4A; Figure S6A‐C). We found that knockout of *Optn* in ESCs leads to accumulation of abnormal mitochondria, as indicated by the existence of increased mitochondrial mass, lower mitochondrial membrane potential, enhanced ROS and decreased ATP generation (Figure 4B‐F). As a result, colony formation and the expression of pluripotency genes were significantly impaired in *optn* ^−/−^ ESCs (Figure 4G, H). The teratoma formation assay was employed to examine the differentiation ability of ESCs. While both *Optn^+/+^* and *o* *ptn* ^−/−^ ESCs formed teratomas, the average weight of teratomas formed by *optn* ^−/−^ ESCs was significantly lower than those formed by *Optn^+/+^* ESCs (Figure 4I, J). The compromised self‐renewal, pluripotency and differentiation in *optn* ^−/−^ ESCs were not due to impaired cell death or proliferation, since *Optn* knockout did not affect cellular proliferation and viability (Figure S6D, E).

In addition, we generated both *ndp52* ^−/−^ and *ndp52* ^−/−^+*optn* ^−/−^ ESCs (Figure S7). Although deletion of *Ndp52* moderately changed mitochondrial homeostasis and self‐renewal of ESCs, knockout of *ndp52* in *optn* ^−/−^ ESCs did not have additive effects (Figure S8).

### 3.5 Regulation of stemness by OPTN depends on TBK1 phosphorylation and ubiquitin binding activity

**FIGURE 5:**
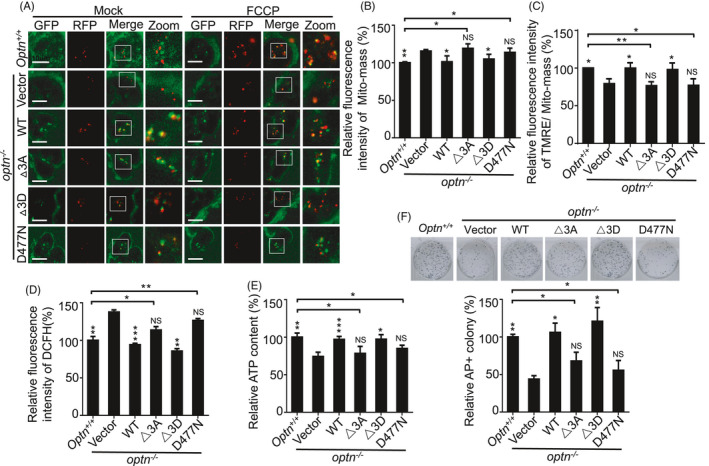
Regulation of pluripotency by OPTN depends on its phosphorylation and ubiquitin binding activity. A, Reduced mitophagosomes in *optn* ^−/−^ ESCs were restored by gain of expression of WT or △3D mutant *Optn*, but not △3A or D477N mutant *Optn*. The FCCP was used to treat cells at 10 nM for 3 h. GFP, Keima(mitochondria) Ex. 440 nm; RFP, Keima(autolysome) Ex. 590 nm. B, Mitochondrial accumulation in *optn* ^−/−^ ESCs was rescued by gain of expression of WT and △3D OPTN, but not △3A and D477N OPTN. Data are shown as mean ± SD, n = 3; *P < .05; **P < .01; NS, not significant; Student's *t* test. C, Abnormal mitochondrial membrane potential in *optn* ^−/−^ ESCs was rescued by gain of expression of WT and △3D OPTN, but not △3A and D477N OPTN. Data are shown as mean ± SD, n = 3; *P < .05; **P < .01; NS, not significant; Student's *t* test. D, Increased ROS in *optn* ^−/−^ ESCs was rescued by gain of expression of WT and △3D OPTN. but not △3A and D477N OPTN. Data are shown as mean ± SD, n = 3; **P < .01; ***P < .001; NS, not significant; Student's *t* test. E, Decreased ATP in *optn* ^−/−^ ESCs was rescued by gain of expression of WT and △3D OPTN, but not △3A and D477N OPTN. Data are shown as mean ± SD, n = 3; *P < .05; **P < .01; ***P < .001; NS, not significant; Student's *t* test. F, Aberrant self‐renewal of *optn* ^−/−^ ESCs was rescued by gain of expression of WT and △3D OPTN, but not △3A and D477N OPTN. Data are shown as mean ± SD, n = 3; *P < .05; **P < .01; NS, not significant; Student's *t* test. Statistical analysis was performed by comparing the values of each individual cell line with the value of *optn* ^−/−^ stable ESC line carrying empty vector respectively. In addition, Statistical analysis was also performed by comparing the value of *optn^+/+^* ESC with the values of *optn* ^−/−^ stable ESC lines carrying constructs expressing △3A or D477N mutant *Optn* respectively

In human somatic cells, mitochondrial depolarization activates the kinase TBK1 to phosphorylate the autophagy receptor OPTN at Serine 177 (S177), Serine 473 (S473) and Serine 513 (S513); this promotes ubiquitin chain binding by OPTN, mitochondrial retention of OPTN and efficient mitophagy.^23^, ^26^ To dissect how OPTN is involved in mitochondrial homeostasis and pluripotency regulation in ESCs, a gain‐of‐function assay was performed by introducing an *Optn* expression vector into *optn* ^−/−^ ESCs. We made OPTN constructs with mutations of the 3 critical TBK1 phosphorylation sites (S187, S476 and S517) to create the activated form of OPTN (S187D, S476D and S517D; △3D) or the inactivated form (S187A, S476A and S517A; △3A). In addition, the ubiquitin‐binding domain‐disrupting mutant OPTN (D477N) was constructed.^26^, ^27^ We then established stable *optn* ^−/−^ ESC lines carrying an empty vector, WT *Optn*, *Optn*(△3D), *Optn*(△3A) or ubiquitin‐binding‐deficient mutant *Optn*(D477N) (Figure S9A, B). The results showed that reduced mitophagosomes, accumulation of abnormal mitochondria, decreased mitochondrial membrane potential, elevated ROS and compromised ATP generation were restored in *optn* ^−/−^ stable ESC lines carrying WT or △3D *Optn* but not in those with the empty vector, △3A or D477N *Optn* (Figure 5A‐E). These data support the idea that TBK1 phosphorylation and ubiquitin binding are critical for OPTN‐mediated mitophagy in ESCs. Correspondingly, the defective colony formation ability was rescued in *optn* ^−/−^ stable ESC lines carrying wild‐type and △3D *Optn* (Figure 5F). Collectively, these data demonstrate that OPTN regulates ESC stemness in a manner that depends on TBK1 phosphorylation and ubiquitin‐binding activity.

### 3.6 PINK1 mediates mitochondrial homeostasis and stemness through OPTN

**FIGURE 6:**
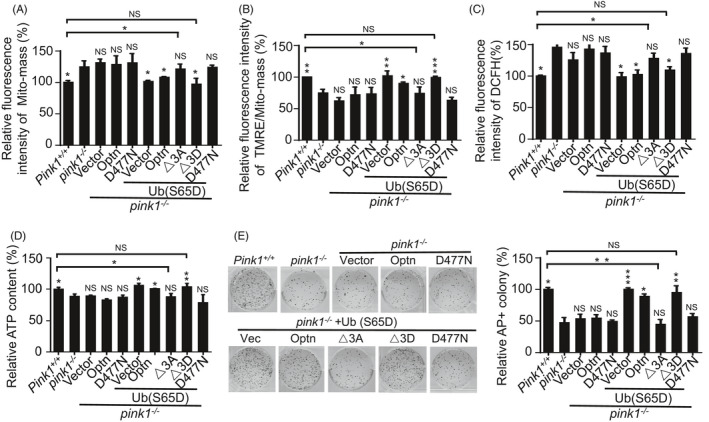
PINK1 regulates mitochondrial homeostasis and pluripotency through OPTN. A, Accumulation of mitochondria in *pink1* ^−/−^ ESCs was rescued by gain of expression of Ub‐S65D only, or Ub‐S65D together with WT or △3D mutant *Optn*. Data are shown as mean ± SD, n = 3; *P < .05; NS, not significant; Student's *t* test. B, Decreased mitochondrial membrane potential in *pink1* ^−/−^ ESCs was rescued by gain of expression of Ub‐S65D only, or Ub‐S65D together with WT or △3D mutant *Optn*. Data are shown as mean ± SD, n = 3; *P < .05; **P < .01; ***P < .001; NS, not significant; Student's *t* test. C, Increased ROS in *pink1* ^−/−^ ESCs was rescued by gain of expression of Ub‐S65D only, or Ub‐S65D together with WT or △3D mutant *Optn*. Data are shown as mean ± SD, n = 3; *P < .05; NS, not significant; Student's *t* test. D, Decreased ATP in *pink1* ^−/−^ ESCs was rescued by gain of expression of Ub‐S65D only, or Ub‐S65D together with WT or △3D mutant *Optn*. Data are shown as mean ± SD, n = 3; *P < .05; **P < .01; NS, not significant; Student's *t* test. E, Aberrant self‐renewal of *pink1* ^−/−^ ESCs was rescued by gain of expression of Ub‐S65D only, or Ub‐S65D together with WT or △3D mutant *Optn*. Data are shown as mean ± SD, n = 3; *P < .05; **P < .01; ***P < .001; NS, not significant; Student's *t* test. Statistical analysis was performed by comparing the values of each individual cell line with the value of *pink1* ^−/−^ stable ESC line carrying empty vector respectively. In addition, statistical analysis was also performed by comparing the value of *pink1^+/+^* ESC with the values of *pink1* ^−/−^ stable ESC line carrying constructs expressing △3A or △3D mutant *Optn* respectively

We next investigated whether PINK1 regulates mitochondrial homeostasis and pluripotency through OPTN. In addition to phosphorylating PARKIN at Serine 65 (S65) to activate its ubiquitin ligase activity, PINK1 directly phosphorylates ubiquitin at Serine 65 (S65).^28^, ^29^ Phosphorylation of ubiquitin by PINK1 enhances OPTN binding and recruitment to mitochondria to promote mitophagy in the absence of PARKIN.^22^ We first generated *pink1* ^−/−^ stable ESC lines carrying either empty vector, or constructs expressing of WT *Optn* or ubiquitin‐binding‐deficient mutant *Optn* (D477N) or phosphomimetic ubiquitin (Serine 65 to Aspartate, Ub‐S65D). Then we transfected an empty vector or constructs expressing WT *Optn*, *Optn*(△3D), *Optn*(△3A) or the ubiquitin‐binding‐deficient mutant *Optn*(D477N) into Ub‐S65D expressing *pink1* ^−/−^ ESCs to establish stable cell lines (Figure S10A). Neither expression of wild‐type or D477N *Optn* in Ub‐S65D deficient *pink1* ^−/−^ ESCs, nor expression of △3A or D477N *Optn* in Ub‐S65D expressing *pink1* ^−/−^ ESCs restored the reduced mitophagosomes, dysfunctional mitochondrial accumulation, decreased mitochondrial membrane potential, enhanced ROS generation and reduced ATP generation. In contrast, gain of expression of WT or △3D *Optn* in Ub‐S65D expressing *pink1* ^−/−^ ESCs, or Ub‐S65D only in *pink1* ^−/−^ ESCs rescued these defects (Figure 6A‐D; Figure S10B). Correspondingly, the compromised self‐renewal ability of *pink1* ^−/−^ ESCs was rescued by gain of expression of Ub‐S65D only or Ub‐S65D together with WT or △3D mutant *Optn* in *pink1* ^−/−^ ESCs (Figure 6E). Together, these data indicate that PINK1 promotes OPTN association with mitochondria by phosphorylation of Ub at S65, and thus regulates mitochondrial homeostasis and stemness.

## 4 DISCUSSION

Pluripotent stem cells have fewer mitochondria than adult somatic cells. Studies by us and others have shown that mitochondria are cleared by autophagy during somatic cell reprogramming.^14^, ^30^, ^31^ In ESCs, damaged mitochondria are degraded by autophagy to maintain mitochondrial homeostasis and pluripotency.^14^, ^18^ However, it remains unclear how damaged mitochondria are degraded by autophagy for pluripotency regulation. Here, we demonstrated that PINK1/OPTN‐mediated PARKIN‐independent mitophagy is responsible for regulation of mitochondrial homeostasis in both acquisition and maintenance of pluripotency, and plays critical roles in ESC differentiation.

Pluripotent stem cells favor glycolysis over oxidative phosphorylation for energy production.^32^ Mitochondria are the sites of the Krebs cycle and oxidative phosphorylation. Whether mitochondria are involved in stemness regulation in pluripotent stem cells (PSCs) is still controversial. An early study showed that inhibition of mitochondrial respiration promotes pluripotency,^33^ while another report suggested that normal mitochondria control proliferation of ESCs but have no effect on ESC pluripotency.^34^ Recently, we have identified that high autophagic flux is an intrinsic characteristic of mouse ESCs that can regulate cellular protein and organelle homeostasis.^18^ Dysfunction of autophagy through *Atg3, Epg5* or *Ulk1* deletion causes decreased protein degradation and accumulation of abnormal mitochondria in ESCs, leading to breakdown of ESC self‐renewal and pluripotency.^14^, ^16^, ^17^ In agreement with these results, studies have shown that activation of both canonical and non‐canonical autophagy is essential for somatic cell reprogramming.^15^, ^31^, ^35^ In contrast, we provide new supporting evidences here that PINK1/OPTN‐mediated selective mitochondrial autophagy is critical for ESC mitochondrial homeostasis and thereafter pluripotency regulation. Our findings consolidate the viewpoint that mitochondria are critical for regulation of ESC stemness.

**FIGURE 7:**
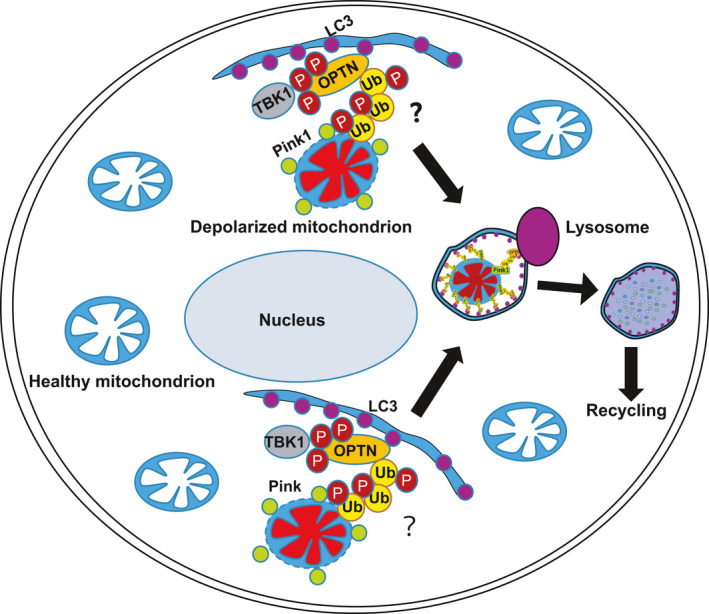
PINK1/OPTN‐mediated mitophagy clears depolarized mitochondria to maintain ESC identity. In ESCs, PINK1 is stabilized on depolarized mitochondria to phosphorylate Ubiquitin and recruit TBK1‐phosphorylated OPTN for autophagic degradation. “?” indicates the unknown E3 ligase

In somatic cells, PINK1 serves as a sensor for the polarization state of mitochondria. Under normal conditions, PINK1 is imported into the mitochondrial intermembrane space and is rapidly degraded by the proteasome.^36^, ^37^ Mitochondrial depolarization inhibits PINK1 import and degradation, leading to accumulation of PINK1 on the OMM.^37^ The accumulated PINK1 phosphorylates ubiquitin at S65 and the ubiquitin‐like domain of PARKIN at S65. The E3 ligase PARKIN is recruited from the cytosol to the OMM, resulting in ubiquitylation of multiple OMM proteins.^28^, ^29^, ^38^, ^39^ The ubiquitylated OMM proteins recruit LIR‐containing autophagy receptors like OPTN for engulfment of the damaged mitochondria, concomitantly with activation of TBK1 kinase.^40^ The activated TBK1 in human cells phosphorylates OPTN at S177, S473 and S513, thus promoting efficient mitophagy.^23^, ^25^, ^26^ In contrast, by using *Pink1*/*Parkin* knockout and mutation of the TBK1 phosphorylation sites on OPTN (S187, S476 and S517) in mouse ESCs, we demonstrated that phosphorylation of OPTN by TBK1 is required for mitophagy, which is independent of PARKIN in ESCs (Figure 7). PINK1 mediated mitochondrial degradation is currently defined to depend on recruitment of ubiquitin to damaged mitochondria by the E3 ligases like PARKIN.^5^ In this regard, the fact that PARKIN is not required for mitochondrial homeostasis and pluripotency regulation indicates the existing undefined E3 ligases in ESCs to build the ubiquitin chains to link depolarized mitochondria with OPTN (Figure 7). This area needs further investigation.

In conclusion, we identified that PINK1/OPTN‐mediated PARKIN‐independent mitophagy serves as a scavenger to degrade depolarized mitochondria in ESCs to maintain mitochondrial homeostasis and pluripotency. These findings enhance our understanding of mitochondrial homeostasis and pluripotency regulation in ESCs, and highlight the importance of mitochondrial function in ESCs.

## CONFLICT OF INTEREST

No potential conflicts of interest were disclosed.

## AUTHOR CONTRIBUTIONS

Chaoqun Wang and Kun Liu designed the project; Chaoqun Wang, Kun Liu, Jiani Cao, Liang Wang, Qian Zhao and Zheng Li performed the experiments; Chaoqun Wang, Kun Liu and Zheng Li analysed the data; Honghai Zhang, Quan Chen and Tongbiao Zhao supervised the experiments; Chaoqun Wang, Kun Liu and Tongbiao Zhao wrote and edited the manuscript. All authors reviewed the manuscript.

## Supporting information

Data S1

## Data Availability

The data that support the findings of this study are available from the corresponding author upon reasonable request.
