# Supplementary material for: PINK1‐mediated mitophagy maintains pluripotency through optineurin
Source: Cell Prolif. 2021 May 1;54(5):e13034. doi: 10.1111/cpr.13034 (PMC8088463; doi:10.1111/cpr.13034)
Supplement: Supplementary file 1 — Data S1 [file CPR-54-e13034-s001.pptx]

## Slide 1
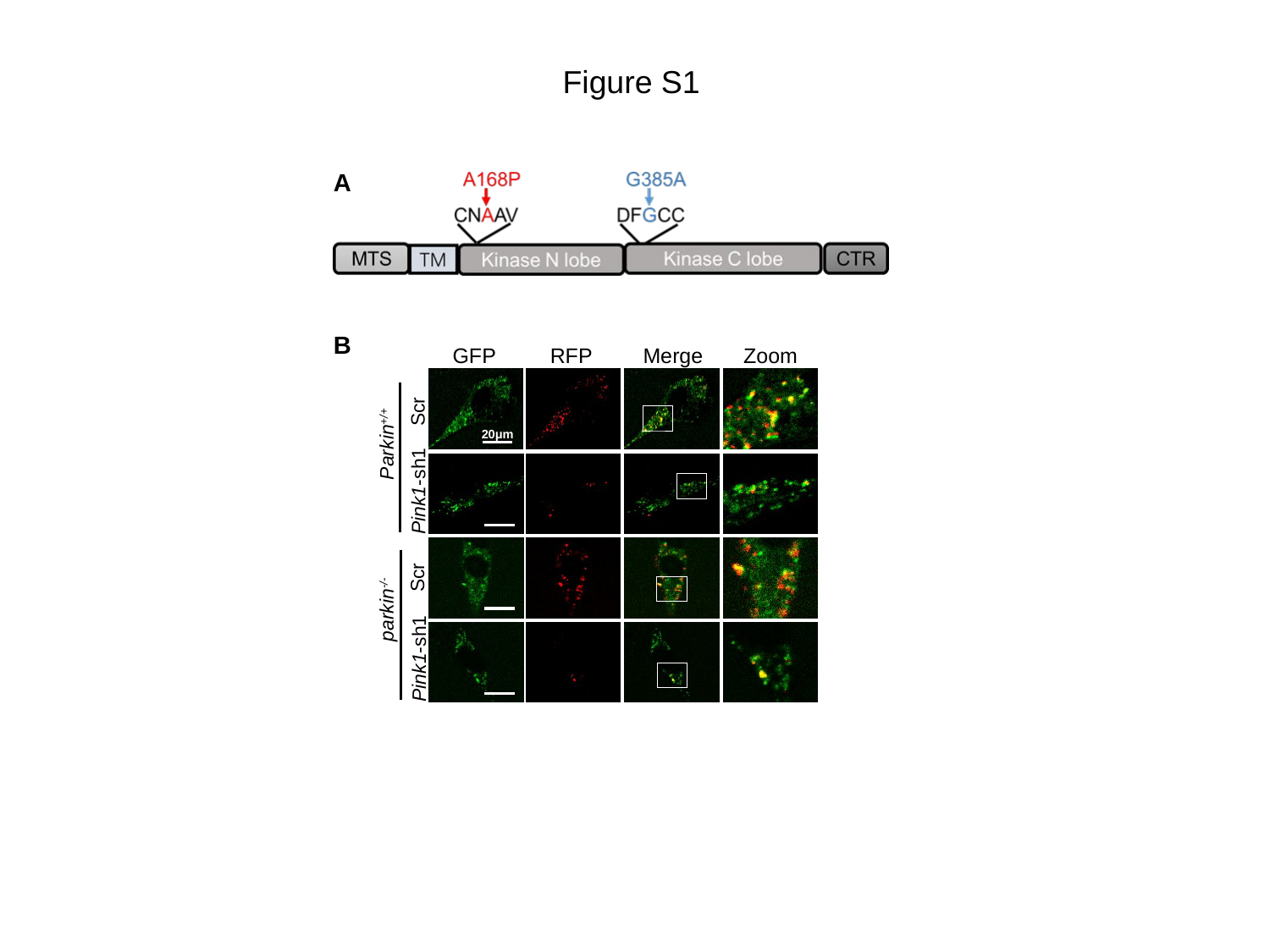

Figure S1
A
B
GFP
RFP
Merge
Zoom
Scr
20μm
Parkin+/+
Pink1-sh1
Scr
parkin-/-
Pink1-sh1

## Slide 2
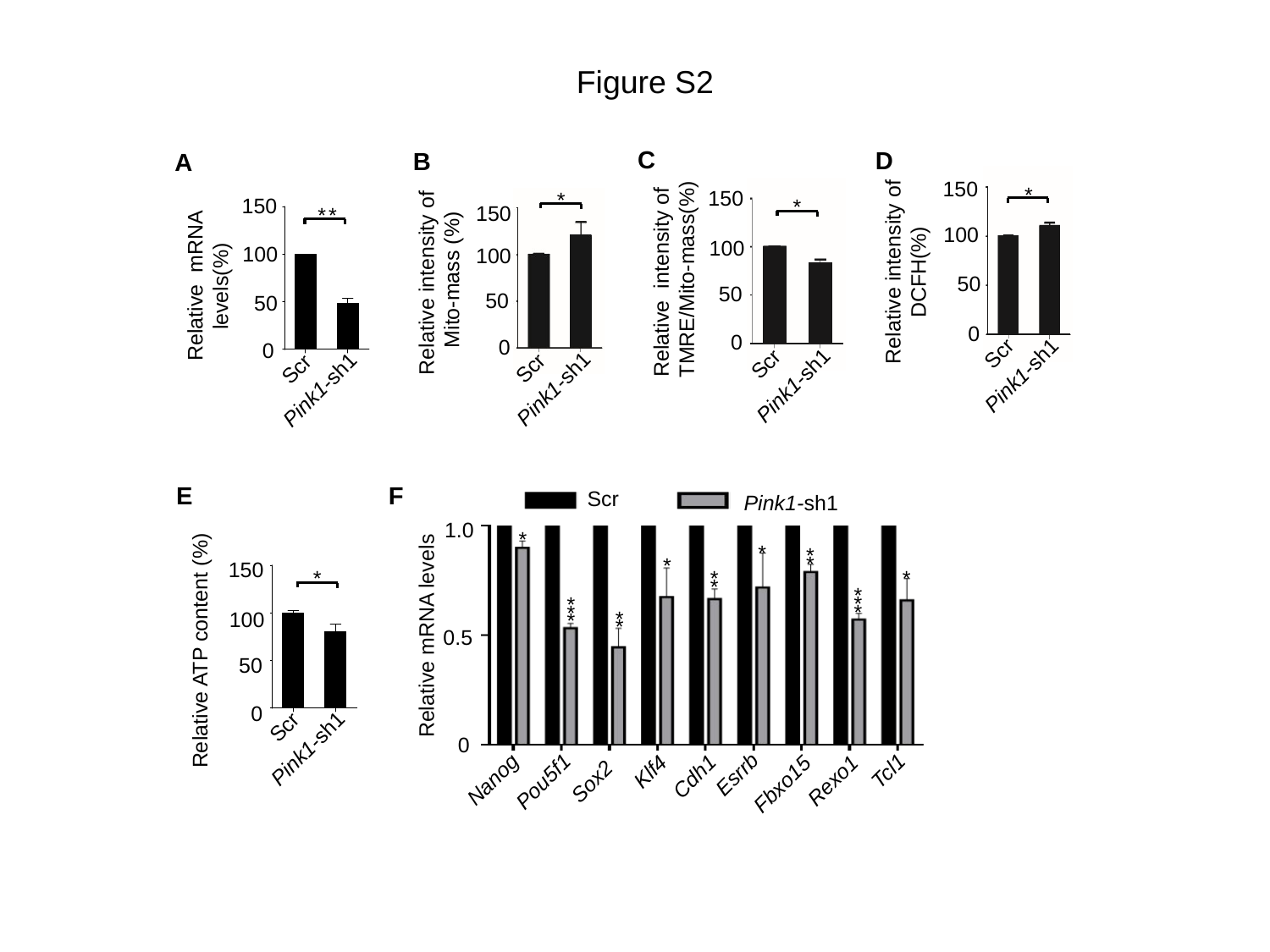

Figure S2
C
D
B
Relative intensity of Mito-mass (%)
 *
150
100
50
0
Scr
Pink1-sh1
A
Relative intensity of DCFH(%)
150
 *
100
50
0
 Scr
Pink1-sh1
Relative intensity of TMRE/Mito-mass(%)
150
 *
100
50
0
Scr
Pink1-sh1
Relative mRNA levels(%)
150
 *
 *
100
50
0
 Scr
Pink1-sh1
E
F
 Scr
Pink1-sh1
1.0
 *
 *
 *
 *
 *
 *
 *
 *
 *
 *
 *
 *
 *
 *
 *
 *
Relative mRNA levels
0.5
0
Tcl1
Klf4
Esrrb
Cdh1
Nanog
Rexo1
Pou5f1
Sox2
Fbxo15
Relative ATP content (%)
150
 *
100
50
0
Scr
Pink1-sh1

## Slide 3
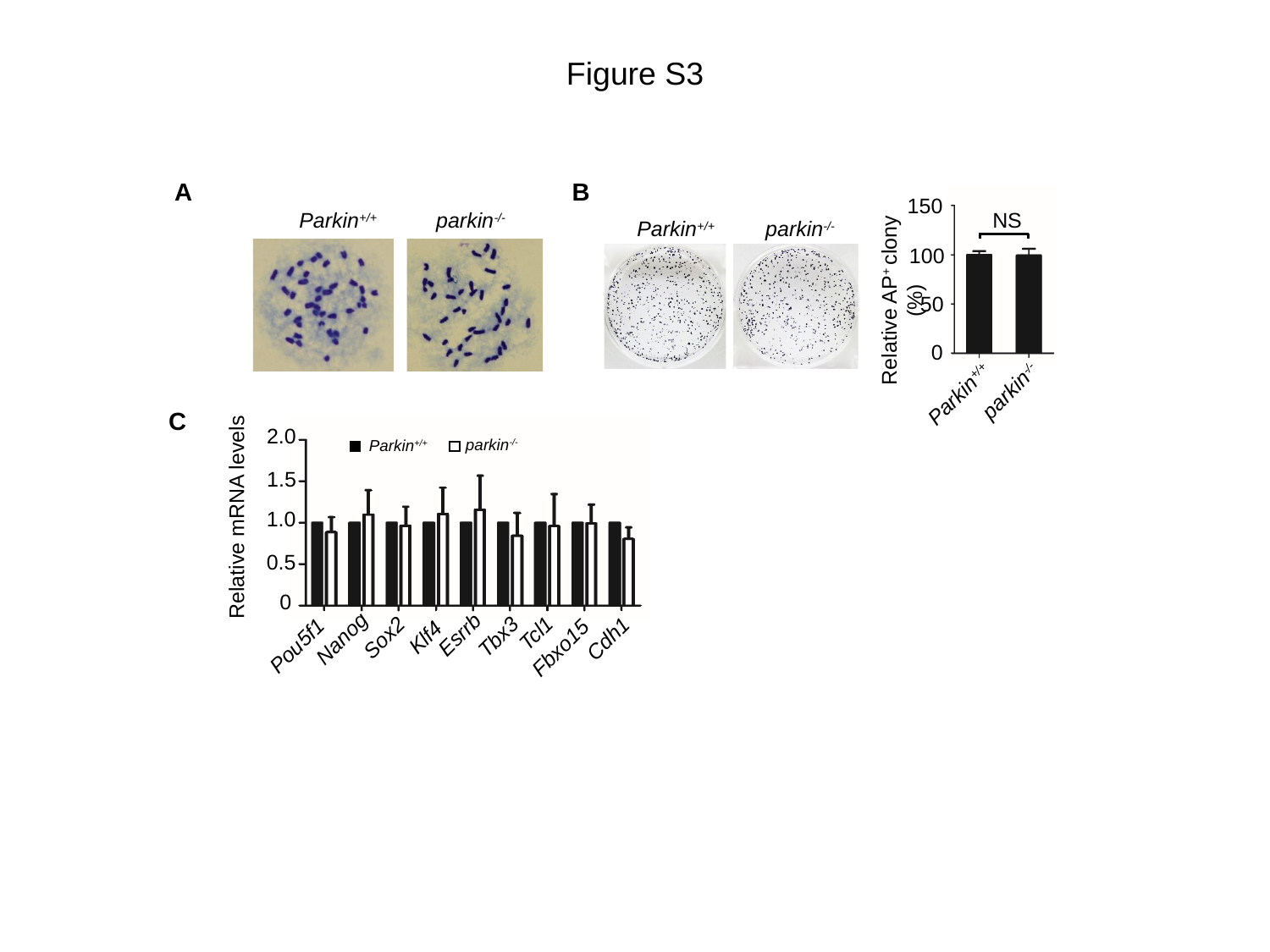

Figure S3
A
B
Relative AP+ clony (%)
150
100
50
0
parkin-/-
Parkin+/+
NS
Parkin+/+
parkin-/-
 Parkin+/+ parkin-/-
2.0
1.5
1.0
0.5
0
Tcl1
Esrrb
Tbx3
Klf4
Sox2
Cdh1
Nanog
Pou5f1
Fbxo15
Relative mRNA levels
parkin-/-
Parkin+/+
C

## Slide 4
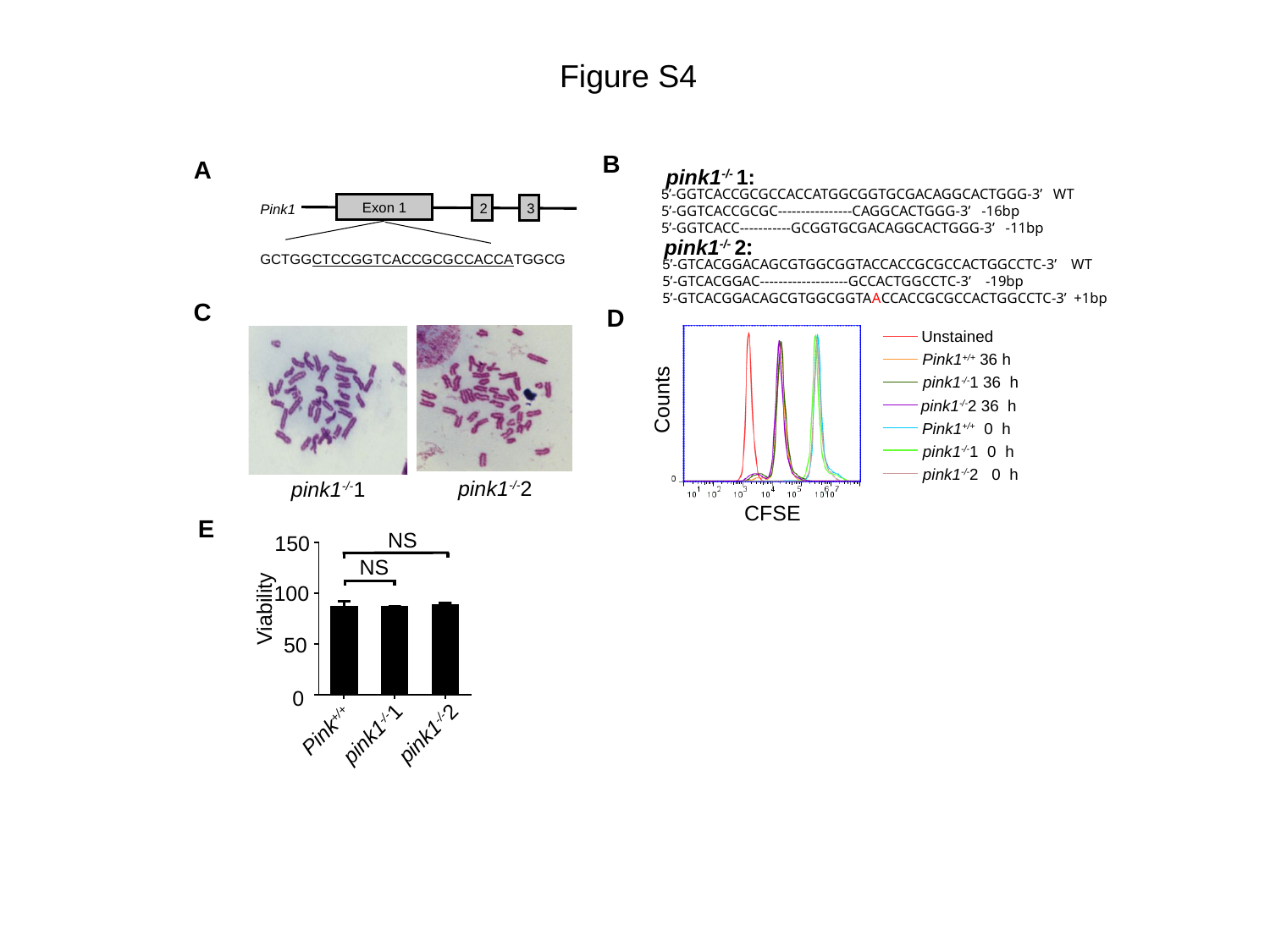

Figure S4
B
A
pink1-/- 1:
5’-GGTCACCGCGCCACCATGGCGGTGCGACAGGCACTGGG-3’ WT
5’-GGTCACCGCGC----------------CAGGCACTGGG-3’ -16bp
5’-GGTCACC-----------GCGGTGCGACAGGCACTGGG-3’ -11bp
Pink1
Exon 1
2
3
GCTGGCTCCGGTCACCGCGCCACCATGGCG
pink1-/- 2:
5’-GTCACGGACAGCGTGGCGGTACCACCGCGCCACTGGCCTC-3’ WT
5’-GTCACGGAC-------------------GCCACTGGCCTC-3’ -19bp
5’-GTCACGGACAGCGTGGCGGTAACCACCGCGCCACTGGCCTC-3’ +1bp
C
D
Unstained
Pink1+/+ 36 h
pink1-/-1 36 h
Counts
pink1-/-2 36 h
Pink1+/+ 0 h
pink1-/-1 0 h
pink1-/-2 0 h
CFSE
pink1-/-2
pink1-/-1
E
NS
150
NS
100
50
0
Pink+/+
pink1-/-1
Viability
pink1-/-2

## Slide 5
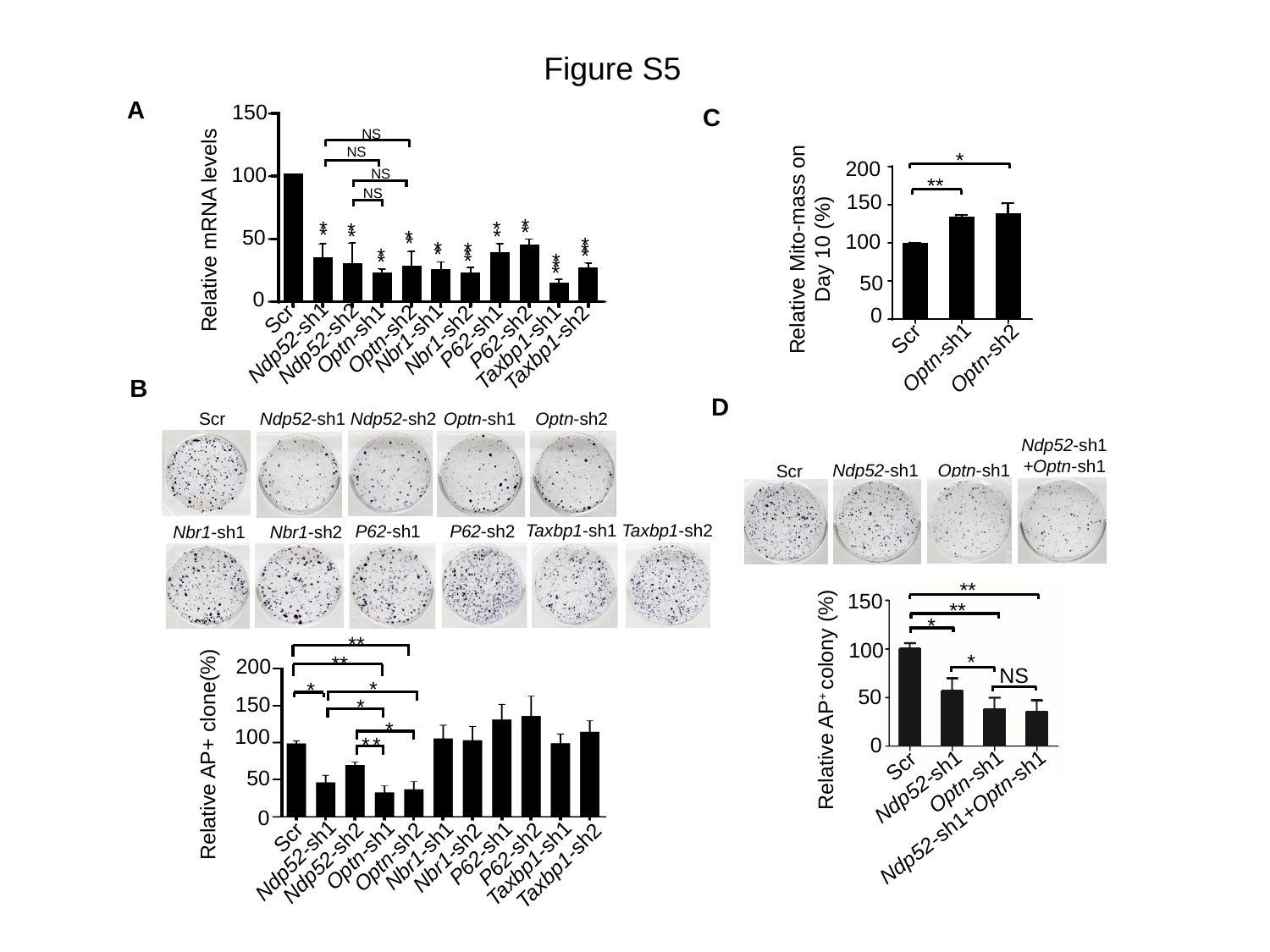

Figure S5
Relative mRNA levels
A
150
 NS
 NS
 NS
100
 NS
 *
 *
 *
 *
 *
 *
 *
 *
50
 *
 *
 *
 *
 *
 *
 *
 *
 *
 *
 *
 *
 *
 *
 *
0
Scr
P62-sh1
Nbr1-sh1
Ndp52-sh2
Optn-sh1
Optn-sh2
Nbr1-sh2
Ndp52-sh1
Taxbp1-sh1
Taxbp1-sh2
P62-sh2
C
Relative Mito-mass on
Day 10 (%)
150
100
50
0
Scr
Optn-sh1
 *
200
**
Optn-sh2
B
D
Optn-sh1 Optn-sh2
Scr Ndp52-sh1 Ndp52-sh2
Taxbp1-sh1 Taxbp1-sh2
P62-sh1 P62-sh2
Nbr1-sh1 Nbr1-sh2
Ndp52-sh1
+Optn-sh1
Optn-sh1
Ndp52-sh1
Scr
Relative AP+ colony (%)
150
100
50
0
 **
 **
 *
 *
NS
Scr
Optn-sh1
Ndp52-sh1
Ndp52-sh1+Optn-sh1
Relative AP+ clone(%)
150
100
50
0
 **
 **
200
 *
 *
 *
 *
 *
 *
Scr
P62-sh1
P62-sh2
Ndp52-sh2
Optn-sh1
Ndp52-sh1
Nbr1-sh1
Optn-sh2
Nbr1-sh2
Taxbp1-sh1
Taxbp1-sh2

## Slide 6
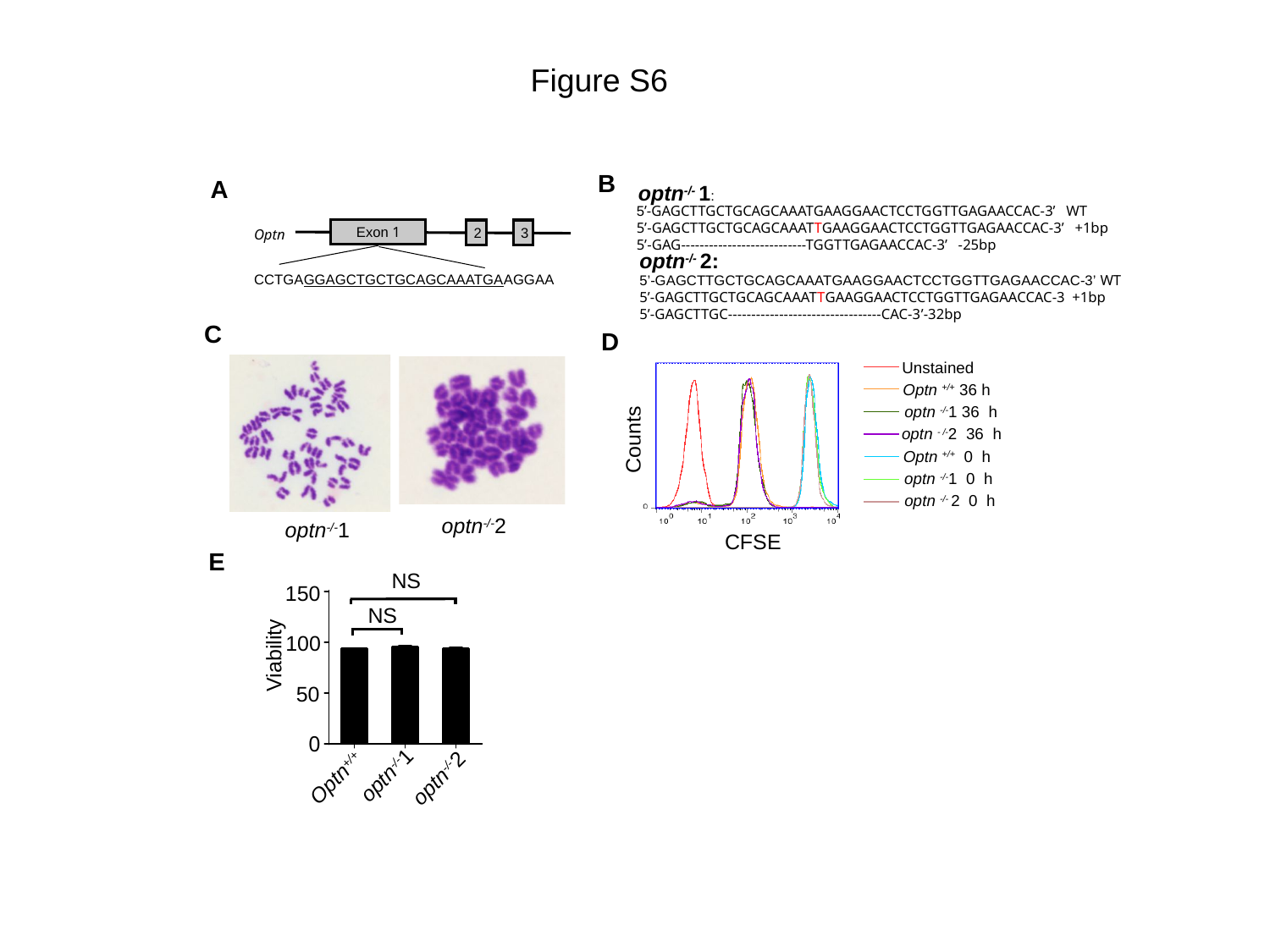

Figure S6
B
A
optn-/- 1:
5’-GAGCTTGCTGCAGCAAATGAAGGAACTCCTGGTTGAGAACCAC-3’ WT
5’-GAGCTTGCTGCAGCAAATTGAAGGAACTCCTGGTTGAGAACCAC-3’ +1bp
5’-GAG---------------------------TGGTTGAGAACCAC-3’ -25bp
optn-/- 2:
5’-GAGCTTGCTGCAGCAAATGAAGGAACTCCTGGTTGAGAACCAC-3’ WT
5’-GAGCTTGCTGCAGCAAATTGAAGGAACTCCTGGTTGAGAACCAC-3 +1bp
5’-GAGCTTGC---------------------------------CAC-3’-32bp
Optn
Exon 1
2
3
CCTGAGGAGCTGCTGCAGCAAATGAAGGAA
C
D
Unstained
Optn +/+ 36 h
optn -/-1 36 h
Counts
optn - /-2 36 h
Optn +/+ 0 h
optn -/-1 0 h
optn -/- 2 0 h
CFSE
 optn-/-2
 optn-/-1
E
NS
NS
100
50
0
optn-/-1
Optn+/+
Viability
optn-/-2
150

## Slide 7
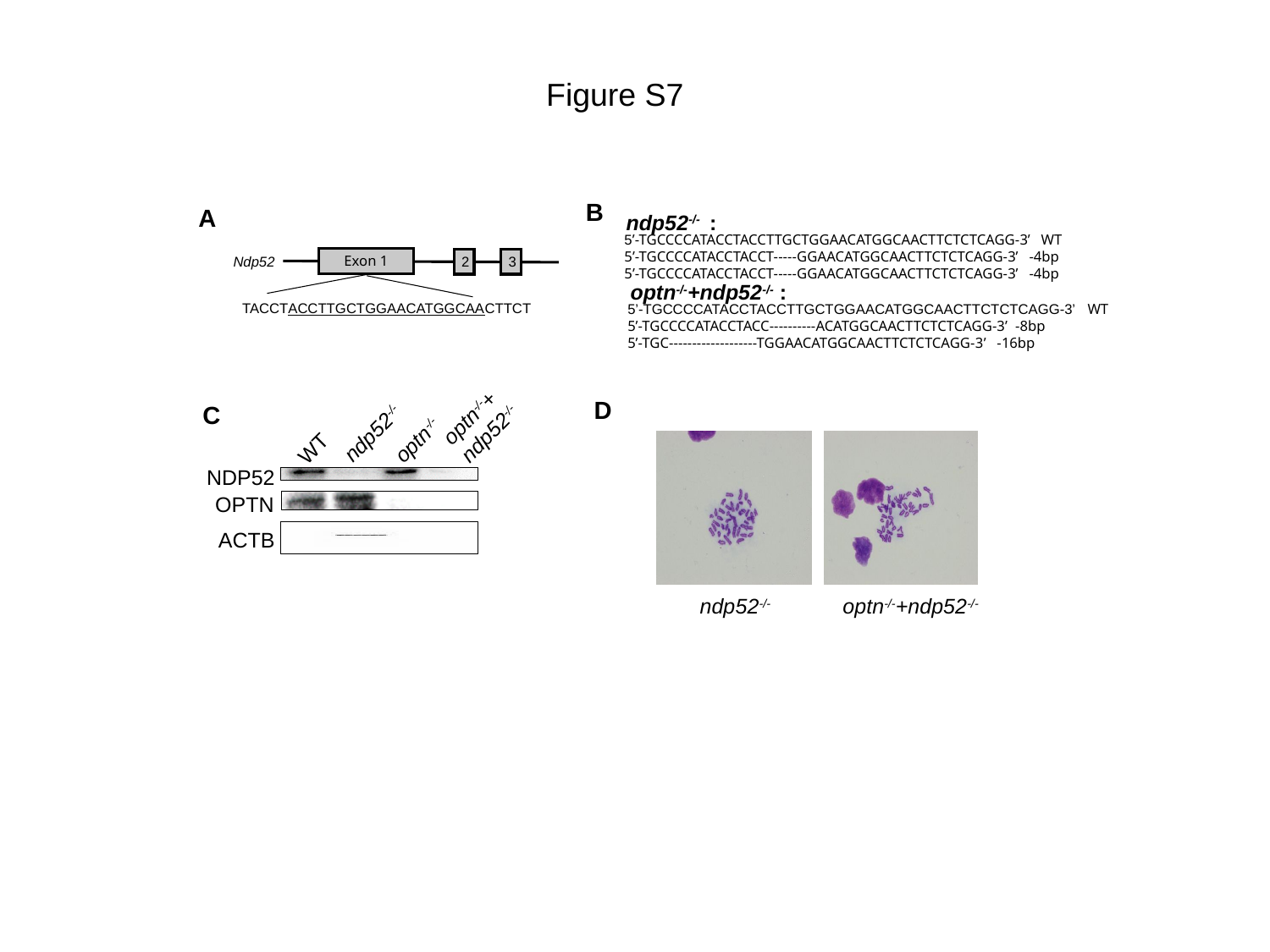

Figure S7
B
A
ndp52-/- :
5’-TGCCCCATACCTACCTTGCTGGAACATGGCAACTTCTCTCAGG-3’ WT
5’-TGCCCCATACCTACCT-----GGAACATGGCAACTTCTCTCAGG-3’ -4bp
5’-TGCCCCATACCTACCT-----GGAACATGGCAACTTCTCTCAGG-3’ -4bp
optn-/-+ndp52-/- :
5’-TGCCCCATACCTACCTTGCTGGAACATGGCAACTTCTCTCAGG-3’ WT
5’-TGCCCCATACCTACC----------ACATGGCAACTTCTCTCAGG-3’ -8bp
5’-TGC-------------------TGGAACATGGCAACTTCTCTCAGG-3’ -16bp
Ndp52
Exon 1
2
3
TACCTACCTTGCTGGAACATGGCAACTTCT
D
 ndp52-/-
 optn-/-+ndp52-/-
optn-/-+
ndp52-/-
ndp52-/-
optn-/-
WT
NDP52
OPTN
ACTB
C

## Slide 8
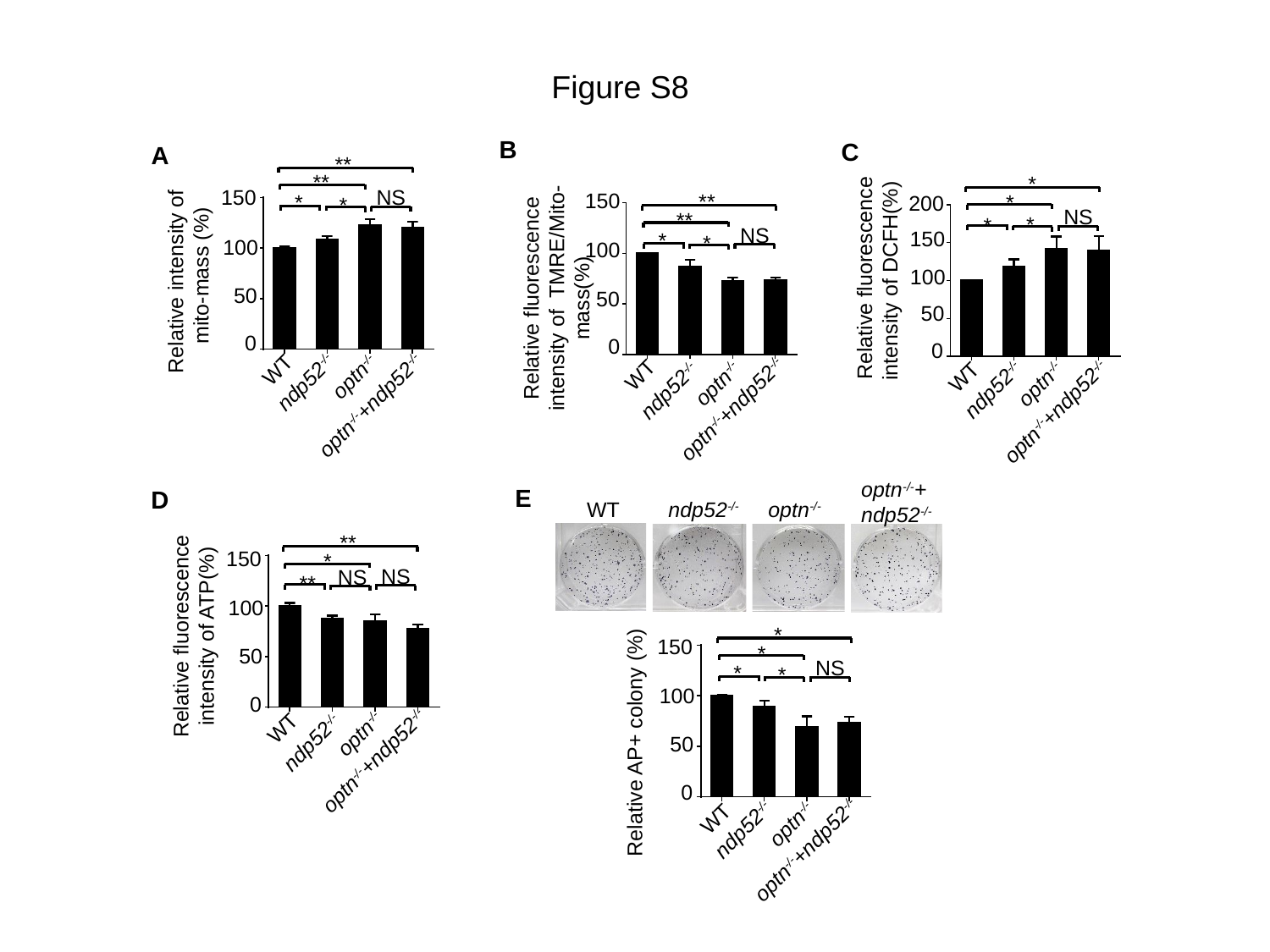

Figure S8
B
Relative fluorescence intensity of TMRE/Mito-mass(%)
150
100
50
0
 **
 **
 NS
 *
 *
WT
optn-/-+ndp52-/-
ndp52-/-
C
 Relative fluorescence intensity of DCFH(%)
150
100
50
0
 *
 *
200
 NS
 *
 *
WT
optn-/-
ndp52-/-
optn-/-+ndp52-/-
A
Relative intensity of
mito-mass (%)
150
100
50
0
 **
 **
 NS
 *
 *
WT
optn-/-
ndp52-/-
optn-/-+ndp52-/-
optn-/-
optn-/-+
ndp52-/-
WT
ndp52-/-
optn-/-
Relative AP+ colony (%)
150
100
50
0
 *
 *
 NS
 *
 *
WT
optn-/-
ndp52-/-
optn-/-+ndp52-/-
E
D
Relative fluorescence intensity of ATP(%)
150
100
50
0
 **
 *
 NS
 NS
 **
WT
optn-/-
ndp52-/-
optn-/-+ndp52-/-

## Slide 9
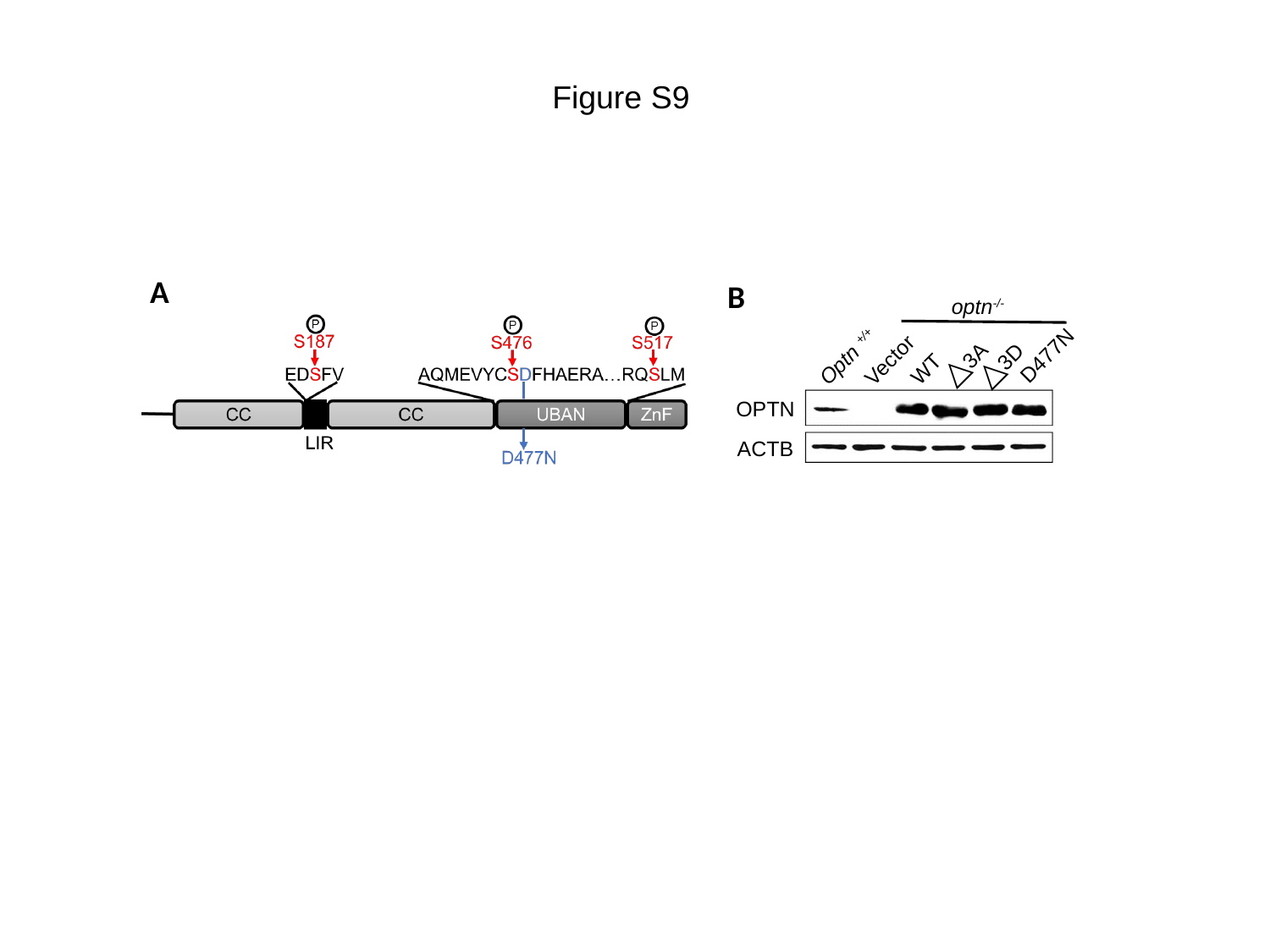

Figure S9
A
B
optn-/-
△3A
D477N
△3D
Optn +/+
Vector
WT
OPTN
ACTB

## Slide 10
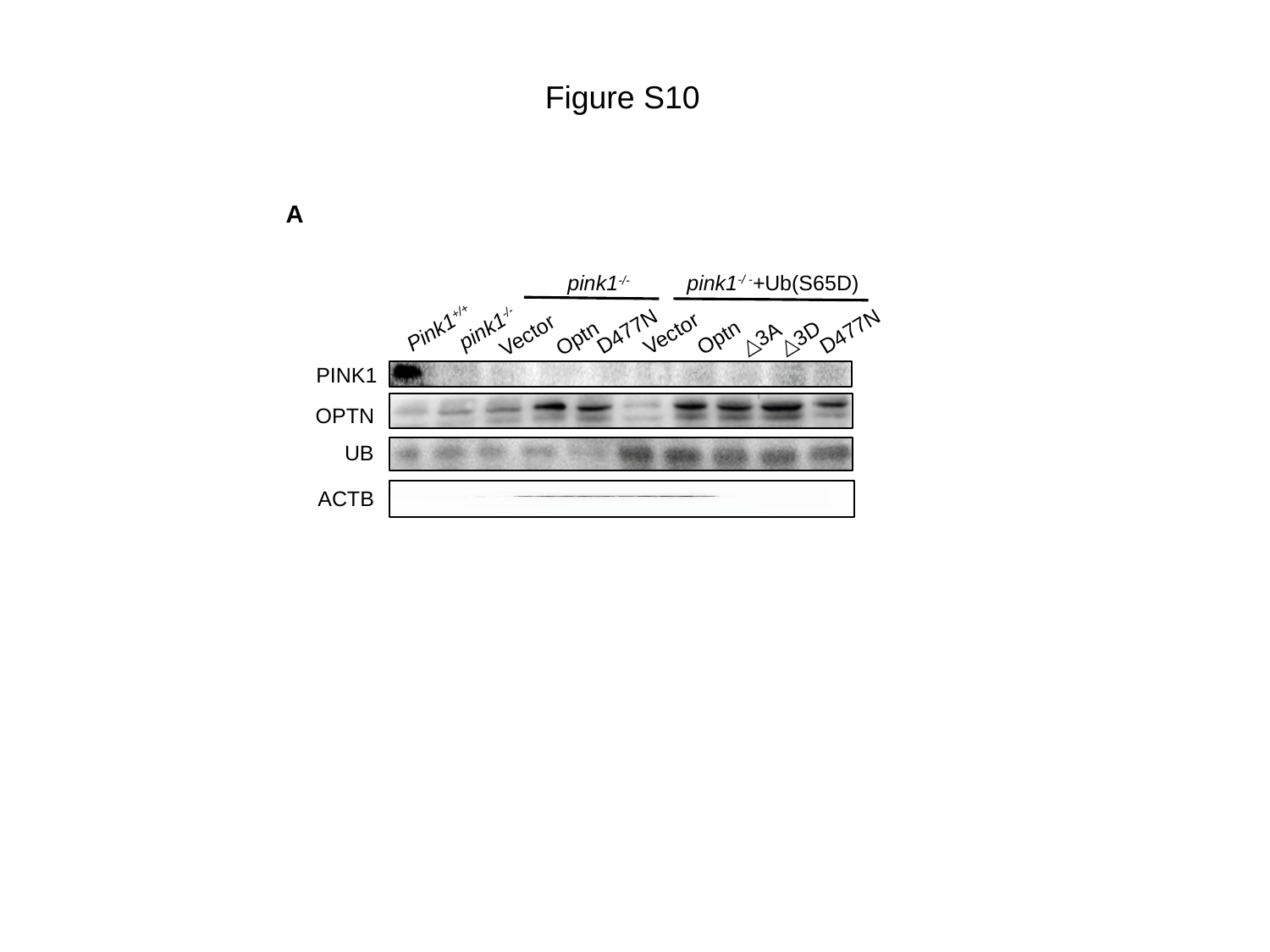

Figure S10
A
 pink1-/ -+Ub(S65D)
pink1-/-
Pink1+/+
pink1-/-
D477N
D477N
Vector
Vector
Optn
Optn
△3D
△3A
PINK1
OPTN
UB
ACTB

## Slide 11
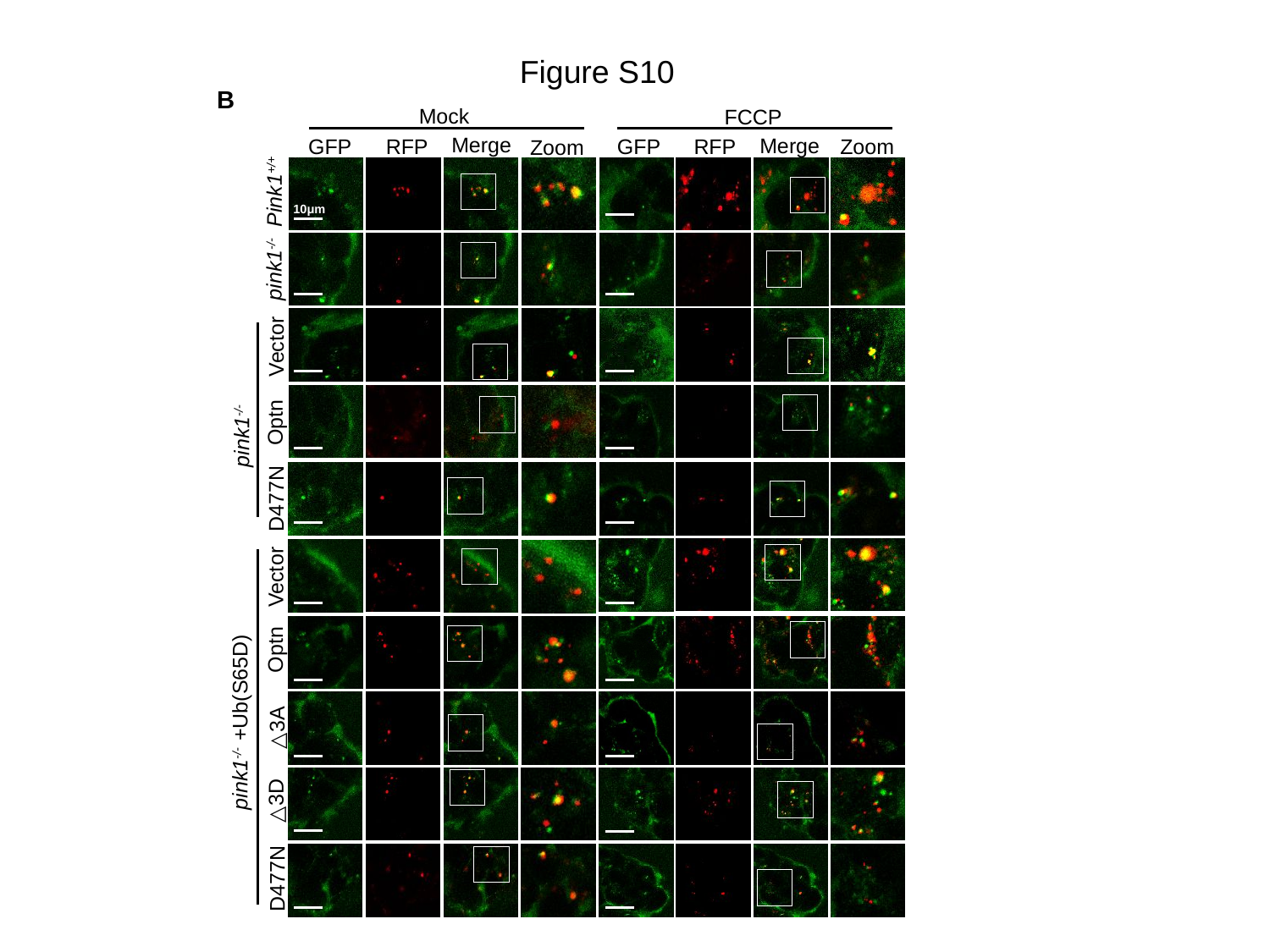

Figure S10
B
Mock
FCCP
Merge
Merge
GFP
Zoom
GFP
RFP
RFP
Zoom
Pink1+/+
10μm
pink1-/-
Vector
Optn
pink1-/-
D477N
Vector
Optn
pink1-/- +Ub(S65D)
△3A
△3D
D477N
